# Effect of Sodium Hypochlorite and Benzalkonium Chloride on the Structural Parameters of the Biofilms Formed by Ten *Salmonella enterica* Serotypes

**DOI:** 10.3390/pathogens8030154

**Published:** 2019-09-17

**Authors:** Rosa Capita, Silvia Fernández-Pérez, Laura Buzón-Durán, Carlos Alonso-Calleja

**Affiliations:** 1 Department of Food Hygiene and Technology, Veterinary Faculty, University of León, E-24071 León, Spain; 2 Institute of Food Science and Technology, University of León, E-24071 León, Spain

**Keywords:** *Salmonella enterica*, serotypes, biocides, biofilms, confocal laser scanning microscopy

## Abstract

The influence of the strain on the ability of *Salmonella enterica* to form biofilms on polystyrene was investigated by confocal laser scanning microscopy. The effects of sodium hypochlorite with 10% active chlorine (SHY; 25,000, 50,000, or 100,000 ppm), and benzalkonium chloride (BZK; 1000, 5000, or 10,000 ppm) on twenty-four-hour-old biofilms was also determined. The biofilms of ten *Salmonella enterica* isolates from poultry (*S.* Agona, *S.* Anatum, *S.* Enteritidis, *S.* Hadar, *S.* Infantis, *S.* Kentucky, *S.* Thompson, *S.* Typhimurium, monophasic variant of *S.* Typhimurium 1,4,(5),12:i:-, and *S.* Virchow) were studied. Biofilms produced by *S.* Anatum, *S.* Hadar, *S.* Kentucky, and *S.* Typhimurium showed a trend to have the largest biovolume and the greatest surface coverage and thickness. The smallest biofilms (*P* < 0.01) in the observation field (14.2 × 10^3^ µm^2^) were produced by *S.* Enteritidis and *S.* 1,4,(5),12:i:- (average 12.9 × 10^3^ ± 9.3 × 10^3^ µm^3^) compared to the rest of the serotypes (44.4 × 10^3^ ± 24.7 × 10^3^ µm^3^). Biovolume and surface coverage decreased after exposure for ten minutes to SHY at 50,000 or 100,000 ppm and to BZK at 5000 or 10,000 ppm. However, the lowest concentrations of disinfectants increased biovolume and surface coverage in biofilms of several strains (markedly so in the case of BZK). The results from this study suggest that the use of biocides at low concentrations could represent a public health risk. Further research studies under practical field conditions should be appropriate to confirm these findings.

## 1. Introduction

Salmonellosis is the zoonosis associated with the largest number of outbreaks of food-borne disease in the European Union. It comes in second place after campylobacteriosis in terms of numbers of sporadic cases. Specifically, in 2017, *Salmonella enterica* was responsible for 1241 outbreaks of disease in the E.U. (with a total of 9600 associated instances). This was a figure that amounted to 24.4% of the 5079 outbreaks of food-borne disease, with products of animal origin being those most often involved in the infection. The number of confirmed cases of human salmonellosis in 2017 came to 91,662 (the notification rate was 19.7 cases per 100,000 inhabitants). The rate of admissions to hospital (calculated by using the number of cases of known status) was 42.5%, and the mortality rate was 0.25% [1]. Similarly, this is the pathogenic microorganism involved in the largest number of notifications passed to the Rapid Alert System for Food and Feed of the E.U. (RASFF). In 2017, Member States submitted 207 notifications relating to *Salmonella* out of a total of 414 notifications covering all pathogenic microorganisms. Third Countries submitted 471 notifications in respect of this microorganism out of a total of 561 notifications relating to pathogenic microorganisms [2]. 

The main serotypes of *Salmonella* implicated in human infections in the E.U. are *Salmonella enterica* serotype Enteritidis and *Salmonella enterica* serotype Typhimurium. In 2017 the first of these was involved in 49.1% and the second in 13.4% of cases of illness [1]. Other serotypes that are frequently concerned in human salmonellosis include *Salmonella enterica* serotype Agona, *Salmonella enterica* serotype Derby, *Salmonella enterica* serotype Hadar, *Salmonella enterica* serotype Infantis, *Salmonella enterica* serotype Kentucky, *Salmonella enterica* serotype Newport, *Salmonella enterica* serotype 1.4.[5].12:i:- (monophasic variant of *S.* Typhimurium), *Salmonella enterica* serotype Thompson, and *Salmonella enterica* serotype Virchow [1,3].

*Salmonella enterica* has the ability to form biofilms. These are complex microbial communities set in an extracellular polymer matrix produced by the bacteria themselves and adhering firmly to inert surfaces and living tissue, constituting the predominant form of microbial growth in nature. Cells in biofilms have a considerably increased resistance to environmental challenges, including disinfectants and antibiotics, relative to planktonic cells. This is the reason that biofilms have been identified as a major factor in the persistence of food-borne pathogens in food-processing environments [4]. Biofilms have been shown to be the main source for the contamination of foodstuffs and have been associated with more than 60% of outbreaks of food-borne illness, thus having become a significant problem in the food industry [5]. In addition, serious engineering problems also arise because of the presence of biofilms on the equipment and in the installations of food-processing plants. 

Several compounds are commonly used as antimicrobials for sanitizing surfaces that come into contact with food [6]. Chlorine-based disinfectants, such as sodium hypochlorite (SHY), are oxidizing compounds that show a broad-spectrum bactericidal activity combined with high efficacy and low cost [7]. SHY is approved for use in the European Economic Area (EEA) and/or Switzerland as disinfectant for food and feed areas (product-type 4) [8]. According to Commission Implementing Regulation (EU) 2017/1273, active chlorine released from sodium hypochlorite is approved as an active substance for use in biocidal product-types 1, 2, 3, 4, and 5 when active chlorine concentration in aqueous solution is ≤ 1800 ppm (i.e., ≤ 18% w/w). Quaternary ammonium compounds, such as benzalkonium chloride (BZK), are cationic surfactants that act by disrupting lipid membrane bilayers, being effective against a number of pathogenic microorganisms [9]. BZK is being reviewed for use as a biocide in the EEA area and/or Switzerland for food and feed area disinfection [10]. For both disinfectants to be effective, they must be used at appropriate concentrations. 

Prior studies have noted major differences between serotypes with regard to their capacity to form a biofilm [11,12]. The effect of several disinfectants on *Salmonella* biofilms has been also assessed [13,14]. Nonetheless, viable counts, crystal violet assay, or scanning electron microscopy are used to study biofilms in most reports, and it would seem that research works quantitatively assessing the effect of different concentrations of SHY and BZK on structural parameters (e.g., biovolume, surface coverage, thickness, or roughness) of biofilms formed by different *Salmonella* serotypes are lacking. Thus, there is a need for further research that would allow quantitative comparisons of the three-dimensional structures and the structural parameters of biofilms of various serotypes of *Salmonella* before and after exposure to different concentrations of several disinfectants. The aim of this research work was to compare, by means of confocal laser scanning microscopy (CLSM) and quantitative image analysis, the structural parameters of biofilms formed by strains belonging to ten different serotypes of *Salmonella* and to determine the effectiveness of various concentrations of two food-grade biocides (sodium hypochlorite and benzalkonium chloride) on these structures. The ultimate objective was to identify the concentrations that were fittest for this purpose.

## 2. Results and Discussion

### 2.1. Architecture of Salmonella enterica Biofilms 

**Figure 1:**
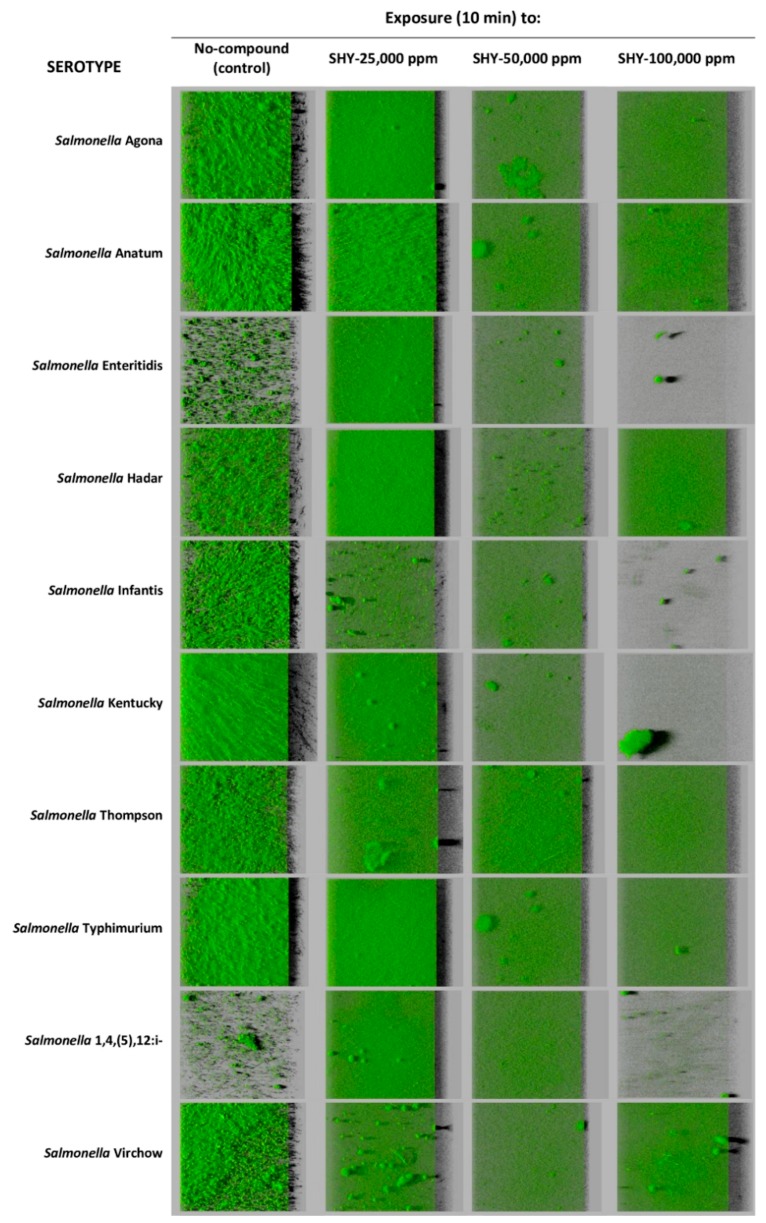
Three-dimensional projections of biofilms formed by ten strains of *Salmonella enterica* before and after exposure to sodium hypochlorite (SHY) at various concentrations. These were obtained from optical sections one micron in size along the *z* axis using a confocal laser scanning microscope. Virtual projections of the shadow to the right are shown, with each square indicated representing a side of 119 μm in length.

All strains of *S. enterica* formed biofilm on polystyrene microtiter plates after 24 h of incubation at 37 °C. The untreated control biofilms presented values varying between serotypes in respect of biovolume in the observation field of 14.2 × 10^3^ µm^2^ (running from 8.2 × 10^3^ ± 1.3 × 10^3^ to 76.5 × 10^3^ ± 34.1 × 10^3^ µm^3^; Table 1), percentage of surface coverage (from 29.1 ± 2.8% to 96.9 ± 3.8%; Table 2), maximum thickness (between 15.7 ± 2.1 and 53.3 ± 13.8 µm; Table 3), and roughness (going from 0.442 ± 0.060 to 0.682 ± 0.048; Table 4). The serotypes *S.* Kentucky and *S.* Hadar showed a trend to produce biofilms with the greatest biovolume, with values in the field observed (14.2 × 10^3^ µm^2^) of 76.5 × 10^3^ ± 34.1 × 10^3^ and 71.8 × 10^3^ ± 24.8 × 10^3^ µm^3^, respectively. These were followed (*P* > 0.05) by *S.* Anatum (54.0 × 10^3^ ± 11.7 × 10^3^ µm^3^) and *S.* Typhimurium (47.5 × 10^3^ ± 6.3 × 10^3^ µm^3^). Biofilms of these four serotypes also showed a trend to have the highest percentage of surface coverage (between 88.9 ± 6.2% and 96.9 ± 3.8%) and the greatest thickness (running from 34.7 ± 4.5 to 53.3 ± 13.8 µm). *S.* Enteritidis and *S.* Typhimurium monophasic variant 1,4,(5),12:i:- showed lower figures (*P* < 0.01) for biovolume, percentage of surface covered, and maximum thickness (12.9 × 10^3^ ± 9.3 × 10^3^ µm^3^, 32.4 ± 6.8%, and 18.3 ± 4.3 µm, respectively, as average) than the rest of the serotypes (44.4 × 10^3^ ± 24.7 × 10^3^ µm^3^, 83.2 ± 12.1%, and 34.6 ± 12.2 µm, respectively). *S.* Enteritidis and *S.* 1,4,(5),12:i:-, unlike the others serotypes, formed only microcolonies of non-confluent cells after twenty-four hours of incubation at 37 °C (Figure 1).

The fact that all strains of *S. enterica* studied had the ability to produce biofilm on polystyrene surfaces is a cause for concern in the context of food safety and public health. This is because the plastic in question is material in very wide use on cattle farms, in slaughterhouses, in food-processing plants, and in establishments serving food. It is frequently used to manufacture a range of surfaces such as piping, cutting boards, and other equipment [15,16]. Moreover, a number of researchers have demonstrated that there is a positive correlation between the production of biofilm on polystyrene microtiter plates and the formation of biofilms on various materials used for surfaces in the food industry [17,18].

The formation of biofilms is one of the commonest strategies used by bacteria in tolerating various sorts of environmental stress [19]. Biofilms increase microbial resistance to physical, biological and chemical agents (for example, antimicrobials), so that the capacity to form biofilms contributes greatly to the persistence of bacteria in food-processing installations [18,20]. Indeed, molecular techniques have been used to show that certain strains of *Salmonella* can remain for several years in food-processing plants [21]. Furthermore, biofilms that are formed in food-processing environments pose a major problem for the food industry, as these structures have been identified as the principal source of contamination of foodstuffs with pathogenic and spoilage microorganisms. This fact constitutes a challenge for public health and involves considerable financial losses [22]. For instance, on these lines it has been shown that the percentage of poultry carcasses contaminated with *Salmonella* grows significantly during processing, as a consequence of the presence of bacteria on surfaces with which these foods come into contact [23].

The present study found striking differences between serotypes with regard to their capacity to form biofilm, as had also been observed previously [11,12,18]. *S.* Kentucky and *S.* Hadar formed strong biofilms (high values for biovolume, percentage of surface covered, and maximum thickness). The fact that *S.* Kentucky is a powerful producer of biofilm is particularly noteworthy, as this serotype is characterized by normally having a high level of resistance to antibiotics of clinical importance, such as ciprofloxacin [24]. The results of the research being reported here do not coincide completely with the findings from previous investigations, in which it was observed that *Salmonella* Hadar (strain SH174) had only a slight ability to form biofilm on polystyrene after 24 h of incubation (15.3 × 10^3^ ± 4.7 × 10^3^ µm^3^ in the observation field of 14.2 × 10^3^ µm^2^) [25]. Similarly, it is not in agreement with the observations of other researchers indicating that *S.* Kentucky and *S.* Hadar are moderate producers of biofilm [26]. These discrepancies among research may be due to variations between different strains of the same serotype with regard to their abilities to form biofilm [11].

The strain of *S.* Typhimurium tested was a strong producer of biofilm. The considerable capacity of strains of this serotype to form biofilm on polystyrene has also been highlighted in previous work [27] in which, after 24 h of incubation on glass at 37 °C, biofilms with a biovolume of 129.4 × 10^3^ ± 34.7 × 10^3^ µm^3^ were seen in the field of observation (14.2 × 10^3^ µm^2^) for another strain of *S.* Typhimurium (strain S175). The great ability to form biofilm that was seen in *S.* Typhimurium in the present study is a finding of considerable interest from the viewpoint of public health, as this serotype is among the most dangerous to humans. Sarwari et al. [28] developed a mathematical model for predicting the capacity to cause human illness, and *Salmonella* Typhimurium attained the highest score of the seven serotypes compared [29]. On the other hand, some authors have noted that the strains of this serotype produce little biofilm on microtiter plates and do so slowly [18]. 

Several researchers have demonstrated that strains of *Salmonella* Agona have a substantial capacity to form biofilm [11,30]. However, the strain of *S.* Agona trialed in the present work was among those producing the least biofilm. Finally, strains of the serotypes *S.* Infantis, *S.* Virchow, and *S.* Enteritidis are, in general, poor at producing biofilm [31], this fact providing backing for some of the findings of the study being reported here. 

### 2.2. Effect of Differing Concentrations of SHY and BZK on Biofilms of Salmonella enterica

Biofilms of *Salmonella* formed on polystyrene after 24 h of incubation at 37 °C were exposed for 10 min to aqueous solutions of SHY (25,000, 50,000, or 100,000 ppm) or BZK (1000, 5000, or 10,000 ppm). 

Use of SHY at 25,000 ppm (2500 ppm of active chlorine) did not reduce the biovolume or the surface coverage of the biofilms in any case. Indeed, for some strains (*S.* Enteritidis and *S.* 1,4,(5),12:i-) this treatment was even associated with an increase in biovolume and in the percentage of surface coverage of the biofilm (Table 1 and Table 2, Figure 1). Even though additional research studies are needed to support these findings, our results suggest that the concentrations of SHY habitually used in disinfecting equipment and installations (800 to 2000 ppm of active chlorine) [9,32] could fail to be effective in eliminating biofilms of *Salmonella* when the disinfectant is applied for a ten-minute period. On these lines, Holah [33] indicated that exposure times in excess of ten minutes are advisable when disinfecting utensils or equipment, especially when they are hard for disinfectants to reach. Furthermore, it should be noted that there are circumstances in which disinfectants are applied in small doses, for instance as the consequence of an inaccurate calculation of concentrations, inappropriate storage of the chemicals, the difficulty of reaching certain locations, or the presence of excessive quantities of organic matter, which reduces the effectiveness of some biocides, such as chlorine compounds [34]. When SHY was applied at 50,000 or 100,000 ppm (5000 or 10,000 ppm of active chlorine, respectively), reductions in biovolume and in the percentage of surface covered by the biofilm were observed relative to the control samples in the majority of cases (Table 1 and Table 2, Figure 1).

**Figure 2:**
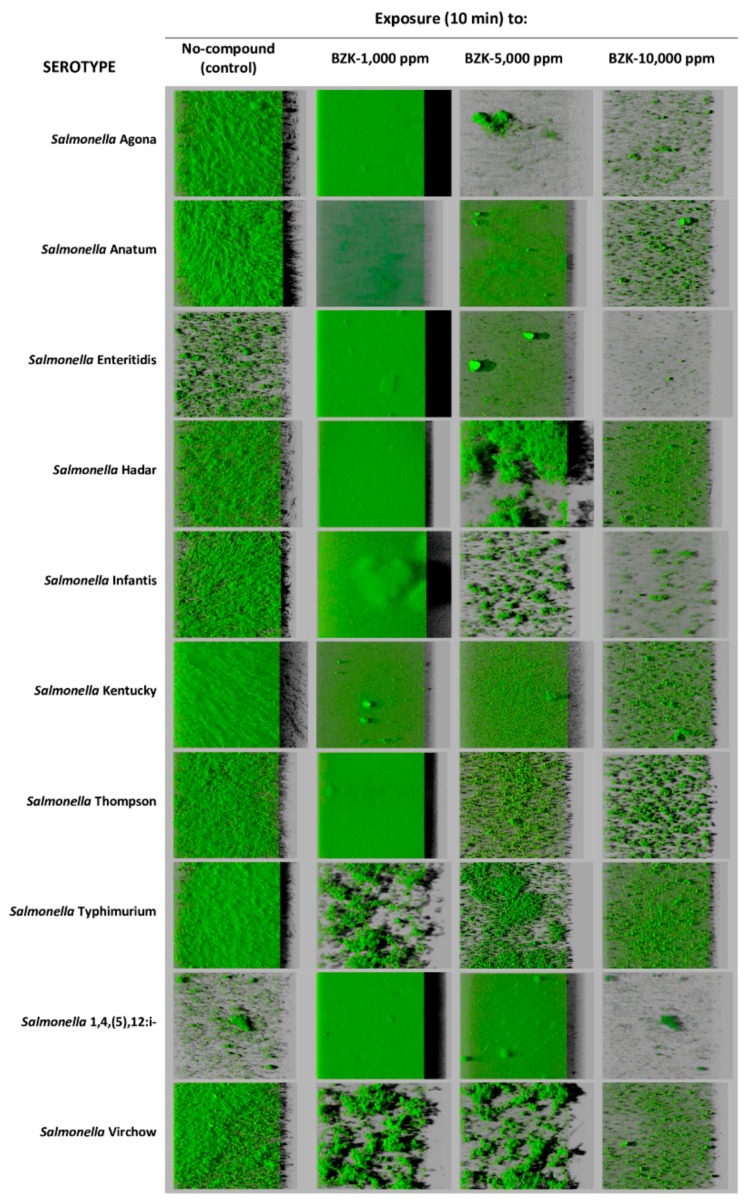
Three-dimensional projections of biofilms formed by ten strains of *Salmonella enterica* before and after exposure to benzalkonium chloride (BZK) at various concentrations. These were obtained from optical sections one micron in size along the *z* axis using a confocal laser scanning microscope. Virtual projections of the shadow to the right are shown, with each square indicated representing a side of 119 μm in length.

One noteworthy feature of this work is the increase in biomass observed after exposure to BZK at 1000 ppm. This treatment caused a significant growth in the biovolume of films relative to unexposed strains in *S.* Agona, *S.* Enteritidis, *S.* Thompson, and *S.* 1,4,(5),12:i:- (Table 5, Figure 2). BZK at 1000 ppm brought about an increase (*P* < 0.05) in percentage of surface covered in *S.* Agona, *S.* Enteritidis, *S.* Infantis, *S.* Thompson, and *S.* 1,4,(5),12:i:- (Table 6, Figure 2). This treatment also caused a marked growth in the maximum thickness of the biofilm in the case of *S.* Agona, *S.* Enteritidis, *S.* Infantis, and *S.* 1,4,(5),12:i-, while the maximum thickness of the film diminished in strains of the serotypes *S.* Anatum and *S.* Typhimurium (Table 7, Figure 2). 

An increased ability to form biofilm in the presence of low doses of SHY or BZK has been demonstrated previously for strains of *Escherichia coli* [34], methicillin-resistant *Staphylococcus aureus* (MRSA) [6], *Salmonella* [27], and *Listeria monocytogenes* [35]. In the present study the biofilms were exposed to disinfectants for ten minutes, and the biocides were then eliminated, even though residual quantities probably remained in the wells of the microtiter plate. A period of two to four hours elapsed between treatment with biocides and microscopic observation, a period during which the exposed strains (in contact with residual amounts of biocides) were able to synthesize biofilm to a greater extent than unexposed strains. The explanation for this greater production of biofilm by the strains exposed to residual doses of SHY or BZK may have to do with the adaptational response of the bacteria, appearing as changes to the structure, composition, or speed of growth of the bacterial cells or as an increase in the extracellular polymer matrix. The part that may be played by certain specialized cellular structures (for example, fimbriae and pili) in the augmented capacity of bacteria to form biofilm has also been highlighted [6]. Nonetheless, further studies would be necessary to confirm these hypotheses. 

BZK is usually employed at a dosage of 1000 to 5000 ppm [9,36,37]. The increase in the biomass of biofilms after exposure for ten minutes to BZK at 1000 ppm underlines the need to further research into practical applications (i.e., on surfaces and equipment present in food-processing facilities) to substantiate these findings and to determine whether the recommended concentrations of BZK should be revised. This is especially crucial in the case of Gram-negative bacteria, which normally are more resistant to quaternary ammonium products as a consequence of modifications in the permeability of their cell walls or through an increased expression of unspecific efflux pumps [38,39]. 

At concentrations of 5000 and 10,000 ppm, BZK was effective in reducing the biovolume of the biofilms formed by most of the strains of *Salmonella* (Table 5). Exposure to BZK at 5000 ppm reduced the percentage of surface covered relative to the control strains in all cases, with the exception of *S.* Enteritidis and *S.* 1,4,(5),12:i:-. At 10,000 ppm, BZK was efficacious in decreasing the percentage of surface covered by the biofilms of all the strains (Table 6). At 5000 and 10,000 ppm of BZK, the maximum thickness of the biofilms decreased in the case of *S.* Anatum, *S.* Hadar (10,000 ppm), *S.* Kentucky, and *S.* Typhimurium (Table 7). These observations coincide with those in studies undertaken by other researchers, who have noted that the biocides used in the food industry are variable in their effects, which go from virtually total elimination of *Salmonella* cells down to a very slight or almost zero reduction, depending on a series of factors, among which the concentrations at which they are used is prominent [40].

Considerable differences were observed in the behavior of the various serotypes in respect to the surface roughness, or rugosity, of the biofilms after exposure to the disinfectants tested. After treatment with SHY, surface roughness tended to decrease in comparison to the unexposed biofilms in the cases of *S.* Anatum, *S.* Enteritidis, *S.* Hadar, *S.* Infantis, and *S.* 1,4,(5),12:i:-. However, an increase in roughness was observed for biofilms of *S.* Virchow after treatment with 25,000 ppm of SHY and for biofilms of *S.* Agona and *S.* Kentucky after treatment with 100,000 ppm of SHY (Table 4). When the biofilms were exposed to 1000 ppm of BZK, rugosity of biofilms formed by *S.* Anatum, *S.* Enteritidis, *S.* Hadar, *S.* Thompson, and *S.* 1,4,(5),12:1- decreased. After treatment with BZK at 5000 ppm or 10,000 ppm, roughness increased in the cases of the serotypes *S.* Agona (10,000 ppm), *S.* Anatum (10,000 ppm), *S.* Kentucky (10,000 ppm), *S.* Typhimurium, and *S.* Virchow. BZK at 5000 ppm decreased roughness in *S.* Anatum and *S.* 1,4,(5),12:1- biofilms. In the other serotypes investigated, no variations were seen in the rugosity of biofilms (Table 8). 

## 3. Materials and Methods

### 3.1. Salmonella Strains and Disinfectants

Ten strains of *Salmonella enterica* were used, derived from samples of chicken meat (*S.* Agona, *S.* Anatum, *S.* Enteritidis, *S.* Hadar, *S.* Infantis, *S.* Kentucky, *S.* Thompson, *S.* Typhimurium, *S.* 1,4,[5],12:i:-, and *S.* Virchow). The strains were stored frozen at −30 °C in tryptone soya broth (TSB, Oxoid Ltd., Hampshire, United Kingdom) with 20% glycerol (vol/vol). In order to carry out the research work, strains were transferred to tubes of TSB, incubated at 37 °C for twenty-four hours, and then inoculated by streaking on tryptone soya agar plates (TSA, Oxoid). Plates were incubated at 37 °C for twenty-four hours and thereafter stored at 4 °C while the experiments were performed. The trials used two disinfectants: sodium hypochlorite (SHY, with 10% of active chlorine; Sigma-Aldrich, Steinheim, Germany) at concentrations of 25,000, 50,000, and 100,000 ppm (2500, 5000, and 10,000 ppm of active chlorine, respectively) and benzalkonium chloride (BZK; Fluka, Deisenhofen, Germany) at concentrations of 1000, 5000, and 10,000 ppm. These concentrations were selected because it had been previously observed that doses below 2500 ppm of active chlorine and below 1000 ppm of BZK were ineffective to eliminate biofilms in the experimental conditions studied (data not shown). The concentrations tested are similar to or higher than the habitually used doses for application in food contact surfaces, which are 800 to 2000 ppm of active chlorine [9,32] and 1000 to 5000 ppm of BZK [9,36,37]. The solutions were prepared with sterile distilled water under aseptic conditions immediately before the experiments.

### 3.2. Biofilm Formation and Analysis

The strains were inoculated into tubes of TSB, which were incubated for 24 h at 37 °C to obtain cultures with approximately 10^9^ cfu/mL. Three decimal dilutions were then prepared in the same culture broth. Microtiter plates were used (Matrix 96-well polystyrene flat-bottom microplates; Thermo Fisher Scientific, Rochester, NY, USA). A volume of 250 µL (approximately 10^6^ cfu/mL) of the third dilution of the culture to be studied was added to the wells. The plates were incubated for one hour at 37 °C to permit bacterial adhesion. After this time had elapsed, the wells were rinsed with a solution of sodium chloride (150 mM NaCl; Sigma-Aldrich, Steinheim, Germany) so as to eliminate any nonadherent cells. The wells were then topped up with 250 µL of sterile TSB. The plate was incubated for 24 h at 37 °C to allow the development of biofilms. The next step was to rinse the wells once again with sodium chloride solution (150 mM NaCl) and add the various solutions of disinfectants, or sterile distilled water in the case of controls. In all cases, contact time was 10 min. The wells were then rinsed with 250 µL of NaCl 150 mM. For staining with fluorescent dye, a volume of 1.25 µL of SYTO 9 (Invitrogen, Barcelona, Spain) was added to 1000 µL of TSB, and 250 μL of this solution was put into each well. The plate was then incubated in the dark at 37 °C for 20 min to enable fluorescent labeling of the bacteria.

The following procedure was used to acquire images with the aid of a Nikon Eclipse TE 2000-U confocal scanning laser microscope using the EZ-C13.60 program (Nikon Instruments Inc., Melville, NY, USA). All biofilms were scanned at 400 Hz, using a 40× objective lens with a 488 nm argon laser set at 90% intensity. Three stacks of horizontal plane images (512 by 512 pixels, corresponding to an area of 119 by 119 µm) with a z step of 1 µm were acquired for each biofilm, using different areas in the well. Three independent experiments were performed for each condition, on different days. Thus, a total of 630 CLSI images were obtained: 10 strains × 7 treatments (2 chemicals × 3 concentrations + controls) × 3 stacks in each biofilm × 3 experiments.

In the processing of the images, use was made of a previously described method [34]. Three-dimensional images of the biofilms were reconstructed by means of the IMARIS 9.1 program (Bitplane AG, Zurich, Switzerland). The term biovolume refers to the average volume of cells (μm^3^) in the field observed (14.2 × 10^3^ µm^2^) and provides an estimate of the biomass of the biofilm. The percentage of surface covered (%) reflects the effectiveness at colonizing the substrate on the part of the bacterial population. The maximum thickness of the biofilm (μm) was determined directly as a function of the number of optical sections of 1 μm recorded on the *z* axis. Roughness provided a measure of how much the thickness of the biofilm varied and was, thus, an indicator of biofilm heterogeneity [41]. A roughness with a value of zero indicates a biofilm of uniform thickness, while a value close to one describes a patchy biofilm. 

### 3.3. Statistical Analysis

The quantitative structural parameters of the biofilms were compared by means of analysis of variance (ANOVA) techniques, with separation of averages achieved by the Tuckey test, utilizing the Statistica® 8.0 software package (StatSoft Inc., Tulsa, OK, USA). Significant differences were established for a probability level of 5% (*P* < 0.05). 

## 4. Conclusions

The ten serotypes of *Salmonella* tested produced biofilms on polystyrene surfaces, although considerable differences were observed between the various serotypes. The serotypes *S.* Anatum, *S.* Hadar, *S.* Kentucky, and *S.* Typhimurium showed a trend to have the highest figures for biovolume, percentage of surface covered, and maximum thickness, while the serotypes *S.* Enteritidis and *S.* 1,4,(5),12:i:- had the lowest values for these structural parameters. Sodium hypochlorite (with 10% active chlorine; SHY) applied at 50,000 or 100,000 ppm and benzalkonium chloride (BZK) at 5000 and 10,000 ppm were effective in reducing biofilms of most strains of *Salmonella* (decreasing both their biovolume and their surface coverage). Nonetheless, both biovolumes and percentages of surface coverage increased when the biofilms were exposed to 25,000 ppm of SHY or to 1000 ppm of BZK. This situation was especially marked in the case of BZK, which is a worrying fact in the context of food safety and public health. The work being reported here has contributed to the determination of what amounts of disinfectant are appropriate for eliminating the biofilms of different serotypes of *Salmonella enterica*. However, results in this research work should be considered with caution because they derive from laboratory-based experiments. Further studies under more realistic conditions should be performed to confirm these findings. Thus, this research study is not aimed at supporting the use of SHY and BZK in the food chain at concentrations other than those included in the recommended use instructions.

## Figures and Tables

**Table 1:** Biovolume (thousands of µm^3^ in the observation field of 14.2 × 10^3^ µm^2^) of twenty-four-hour-old biofilms of several *Salmonella* strains of food origin after exposure to different concentrations of sodium hypochlorite (SHY; 10% of active chlorine).

SEROTYPE	Strains Exposed to:			
	Nonexposed	SHY-25,000 ppm	SHY-50,000 ppm	SHY-100,000 ppm
***Salmonella* Agona**	23.6 ± 4.6^ab^_ab_	52.2 ± 21.5^b^_bc_	14.2 ± 4.3^a^_a_	16.5 ± 7.6^a^_a_
***Salmonella* Anatum**	54.0 ± 11.7^b^_bcd_	49.8 ± 2.6^b^_bc_	24.4 ± 6.1^a^_ab_	18.2 ± 5.4^a^_a_
***Salmonella* Enteritidis**	17.5 ± 12.2^a^_ab_	47.6 ± 16.1^b^_abc_	13.9 ± 9.2^a^_a_	2.4 ± 0.3^a^_a_
***Salmonella* Hadar**	71.8 ± 24.8^b^_cd_	65.9 ± 21.8^b^_c_	16.3 ± 7.1^a^_ab_	15.4 ± 6.9^a^_a_
***Salmonella* Infantis**	31.7 ± 14.1^b^_abc_	15.8 ± 1.8^ab^_a_	11.8 ± 3.7^a^_a_	3.1 ± 1.1^a^_a_
***Salmonella* Kentucky**	76.5 ± 34.1^b^_d_	30.9 ± 4.7^ab^_ab_	15.8 ± 4.9^a^_a_	11.8 ± 9.6^a^_a_
***Salmonella* Thompson**	27.9 ± 1.5^b^_abc_	31.5 ± 3.6^b^_ab_	25.8 ± 3.9^ab^_ab_	16.9 ± 6.3^a^_a_
***Salmonella* Typhimurium**	47.5 ± 6.3^b^_abcd_	45.8 ± 10.6^b^_abc_	38.2 ±18.0^ab^_c_	15.4 ± 6.9^a^_a_
***Salmonella* 1,4,(5),12:i-**	8.2 ± 1.3^b^_a_	32.9 ± 2.8^d^_abc_	15.6 ± 1.0^c^_a_	3.9 ± 0.5^a^_a_
***Salmonella* Virchow**	22.5 ± 1.0^b^_ab_	23.1 ± 1.3^b^_ab_	13.7 ± 3.0^a^_a_	11.1 ± 5.0^a^_a_

Data (mean ± SD; n = 9) in the same row with no letters in common (superscript) are significantly different (*P* < 0.05). Data in the same column with no letters in common (subscript) are significantly different (*P* < 0.05).

**Table 2:** Percentage of surface coverage of twenty-four-hour-old biofilms of several *Salmonella* strains of food origin after exposure to different concentrations of sodium hypochlorite (SHY; 10% active chlorine).

SEROTYPE	Strains Exposed to:			
	Nonexposed	SHY-25,000 ppm	SHY-50,000 ppm	SHY-100,000 ppm
***Salmonella* Agona**	79.6 ± 6.3^bc^_cd_	96.0 ± 3.4^c^_cd_	61.1 ± 11.6^ab^_ab_	39.2 ± 17.6^a^_bc_
***Salmonella* Anatum**	90.0 ± 5.2^b^_cde_	93.7 ± 3.1^b^_cd_	80.1 ± 5.8^ab^_b_	68.5 ± 9.8^a^_de_
***Salmonella* Enteritidis**	35.7 ± 8.8^b^_a_	95.1 ± 3.6^d^_cd_	55.5 ± 4.3^c^_a_	14.7 ± 3.3^a^_a_
***Salmonella* Hadar**	88.9 ± 6.2^c^_cde_	98.6 ± 1.3^c^_d_	62.2 ± 3.0^a^_ab_	78.0 ±3.2^b^_e_
***Salmonella* Infantis**	61.8 ± 8.9^b^_b_	61.8 ± 5.3^b^_a_	55.0± 12.1^b^_a_	12.8± 1.9^a^_a_
***Salmonella* Kentucky**	96.9 ± 3.8^c^_e_	87.8 ± 3.5^c^_c_	64.7 ± 10.7^b^_ab_	22.3 ± 7.1^a^_ab_
***Salmonella* Thompson**	75.7 ± 6.5^b^_bc_	87.4 ± 2.9^b^_c_	82.4 ± 4.3^b^_b_	53.7 ± 10.5^a^_cd_
***Salmonella* Typhimurium**	94,9 ± 0,4^b^_de_	95.3 ± 3.6^b^_cd_	73.2 ± 7.3^a^_ab_	68.0 ± 8.8^a^_de_
***Salmonella* 1,4,(5),12:i-**	29.1 ± 2.8^b^_a_	89.4 ± 2.3^d^_cd_	66.5 ± 2.7^c^_ab_	21.8 ± 1.7^a^_ab_
***Salmonella* Virchow**	77.5 ± 1.3^c^_bc_	74.3 ± 1.2^bc^_b_	60.5 ± 7.1^a^_ab_	64.6 ± 3.0^ab^_de_

For interpretation, see Table 1.

**Table 3:** Maximum thickness (µm) of twenty-four-hour-old biofilms of several *Salmonella* strains of food origin after exposure to different concentrations of sodium hypochlorite (SHY; 10% active chlorine).

SEROTYPE	Strains Exposed to:			
	Nonexposed	SHY-25,000 ppm	SHY-50,000 ppm	SHY-100,000 ppm
***Salmonella* Agona**	33.3 ± 14.6^a^_abc_	26.0 ± 5.3^a^_a_	16.3 ± 4.5^a^_a_	24.7 ± 8.1^a^_abc_
***Salmonella* Anatum**	34.7 ± 4.5^a^_abc_	32.3 ± 0.6^a^_a_	30.0 ± 11.5^a^_a_	26.0 ± 8.7^a^_abc_
***Salmonella* Enteritidis**	21.0 ± 4.6^a^_ab_	38.7 ± 17.8^a^_a_	20.7 ± 14.4^a^_a_	12.3 ± 3.8^a^_a_
***Salmonella* Hadar**	42.3 ± 2.5^a^_bc_	30.0 ± 15.6^a^_a_	27.0 ± 6.2^a^_a_	41.7 ± 13.7^a^_c_
***Salmonella* Infantis**	25.3 ± 7.5^a^_ab_	29.3 ± 2.1^a^_a_	17.3 ± 4.6^a^_a_	19.7 ± 4.5^a^_abc_
***Salmonella* Kentucky**	35.7 ± 9.2^a^_abc_	29.7 ± 4.7^a^_a_	27.0 ± 11.1^a^_a_	25.7 ± 0.6^a^_abc_
***Salmonella* Thompson**	22.0 ± 2.7^a^_ab_	51.3 ± 4.7^b^_a_	33.7 ± 16.6^ab^_a_	38.0 ± 10.0^ab^_bc_
***Salmonella* Typhimurium**	53.3 ± 13.8^a^_c_	29.3 ± 8.5^a^_a_	33.0 ± 2.7^a^_a_	41.7 ± 13.7^a^_c_
***Salmonella* 1,4,(5),12:i-**	15.7 ± 2.1^a^_a_	27.7 ± 5.9^a^_a_	17.7 ± 4.2^a^_a_	15.7 ± 6.7^a^_ab_
***Salmonella* Virchow**	30.0 ± 10.2^a^_abc_	33.7 ± 8.6^a^_a_	18.7 ± 5.7^a^_a_	15.3 ± 6.1^a^_ab_

For interpretation, see Table 1.

**Table 4:** Roughness of twenty-four-hour-old biofilms of several *Salmonella* strains of food origin after exposure to different concentrations of sodium hypochlorite (SHY; 10% active chlorine).

SEROTYPE	Strains Exposed to:			
	Nonexposed	SHY-25,000 ppm	SHY-50,000 ppm	SHY-100,000 ppm
***Salmonella* Agona**	0.451 ±0.019^a^_ab_	0.400 ± 0.034^a^_ab_	0.430 ± 0.044^a^_a_	0.673 ± 0.119^b^_bc_
***Salmonella* Anatum**	0.586 ± 0.068^b^_abcd_	0.451 ± 0.003^a^_bcd_	0.450 ± 0.047^a^_a_	0.464 ± 0.038^a^_abc_
***Salmonella* Enteritidis**	0.682 ± 0.048^b^_d_	0.414 ± 0.025^a^_abc_	0.430 ± 0.112^ab^_a_	0.258 ± 0.151^a^_a_
***Salmonella* Hadar**	0.644 ± 0.067^b^_d_	0.358 ± 0.035^a^_a_	0.428 ± 0.017^a^_a_	0.516 ± 0.123^ab^_abc_
***Salmonella* Infantis**	0.628 ± 0.031^b^_cd_	0.511 ± 0.019^ab^_de_	0.408 ± 0.056^a^_a_	0.684 ± 0.124^b^_bc_
***Salmonella* Kentucky**	0.442 ± 0.060^a^_a_	0.450 ± 0.019^a^_bcd_	0.437 ± 0.017^a^_a_	0.707 ± 0.024^b^_c_
***Salmonella* Thompson**	0.567 ± 0.089^a^_abcd_	0.476 ± 0.015^a^_cde_	0.448 ± 0.033^a^_a_	0.514 ± 0.149^a^_abc_
***Salmonella* Typhimurium**	0.474 ± 0.071^ab^_abc_	0.411 ± 0.024^a^_abc_	0.530 ± 0.023^b^_a_	0.453 ± 0.007^ab^_abc_
***Salmonella* 1,4,(5),12:i-**	0.609 ± 0.035^b^_bcd_	0.462 ± 0.024^a^_bcd_	0.439 ± 0.008^a^_a_	0.340 ± 0.096^a^_a_
***Salmonella* Virchow**	0.452 ± 0.031^a^_ab_	0.550 ± 0.039^b^_e_	0.434 ± 0.031^a^_a_	0.410 ± 0.030^a^_ab_

For interpretation, see Table 1.

**Table 5:** Biovolume (thousands of µm^3^ in the observation field of 14.2 × 10^3^ µm^2^) of twenty-four-hour-old biofilms of several *Salmonella* strains of food origin after exposure to different concentrations of benzalkonium chloride (BZK).

SEROTYPE	Strains Exposed to:			
	Nonexposed	BZK-1000 ppm	BZK-5000 ppm	BZK-10,000 ppm
***Salmonella* Agona**	23.6 ± 4.6^a^_ab_	177.8 ± 50.5^b^_d_	10.2 ± 1.1^a^_a_	8.2 ± 7.4^a^_ab_
***Salmonella* Anatum**	54.0 ±11.7^b^_bcd_	24.5 ± 9.7^a^_a_	10.2 ± 1.5^a^_a_	11.8 ± 6.5^a^_ab_
***Salmonella* Enteritidis**	17.5 ± 12.2^a^_ab_	121.9 ± 58.3^b^_bc_	13.9 ± 1.7^a^_abc_	4.7 ± 4.2^a^_ab_
***Salmonella* Hadar**	71.8 ± 24.8^c^_cd_	57.3 ± 4.6^bc^_ab_	30.9 ± 4.4^ab^_d_	10.3 ± 0.7^a^_ab_
***Salmonella* Infantis**	31.7 ± 14.1^ab^_abc_	51.6 ± 19.1^b^_ab_	10.5 ± 4.2^a^_ab_	2.9 ± 1.2^a^_a_
***Salmonella* Kentucky**	76.5 ± 34.1^b^_d_	19.8 ± 3.8^a^_a_	11.3 ± 4.0^a^_ab_	9.9 ± 1.8^a^_ab_
***Salmonella* Thompson**	27.9 ± 1.5^b^_abc_	63.0 ± 7.3^c^_ab_	17.2 ± 3.4^ab^_abcd_	14.2 ± 1.5^a^_b_
***Salmonella* Typhimurium**	47.5 ± 6.3^b^_abcd_	14.2 ± 8.8^a^_a_	21.1 ± 3.9^a^_abcd_	14.1 ± 1.3^a^_b_
***Salmonella* 1,4,(5),12:i-**	8.2 ± 1.3^a^_a_	125.7 ± 50.1^b^_bc_	26.9 ± 13.2^a^_cd_	4.1 ± 0.8^a^_ab_
***Salmonella* Virchow**	22.5 ± 1.0^bc^_ab_	16.5 ± 3.4^b^_a_	25.4 ± 3.2^c^_bcd_	8.0 ± 1.0^a^_ab_

For interpretation, see Table 1.

**Table 6:** Percentage of surface coverage of twenty-four-hour-old biofilms of several *Salmonella* strains of food origin after exposure to different concentrations of benzalkonium chloride (BZK).

SEROTYPE	Strains Exposed to:			
	Nonexposed	BZK-1000 ppm	BZK-5000 ppm	BZK-10,000 ppm
***Salmonella* Agona**	79.6 ± 6.3^c^_cd_	100.0 ± 0.0^d^_e_	32.6 ± 4.0^b^_b_	9.8 ± 3.7^a^_ab_
***Salmonella* Anatum**	90.0 ± 5.2^c^_cde_	78.7± 3.5^c^_c_	50.3 ± 3.7^b^_c_	21.8 ± 3.9^a^_bcd_
***Salmonella* Enteritidis**	35.7 ± 8.8^b^_a_	99.5 ± 0.9^c^_de_	31.8 ± 1.4^b^_ab_	6.0 ± 3.3^a^_a_
***Salmonella* Hadar**	88.9 ± 6.2^c^_cde_	98.0 ± 0.6^c^_de_	50.7 ± 4.7^b^_c_	33.4 ± 1.0^a^_d_
***Salmonella* Infantis**	61.8 ± 8.9^b^_b_	90.0 ± 4.1^c^_d_	20.6 ± 5.1^a^_a_	9.4 ± 1.2^a^_ab_
***Salmonella* Kentucky**	96.9 ± 3.8^d^_e_	70.7 ± 6.2^c^_c_	52.2 ± 3.2^b^_c_	26.9 ± 1.4^a^_cd_
***Salmonella* Thompson**	75.7 ± 6.5^c^_bc_	98.4 ± 0.7^d^_de_	36.5 ± 1.8^b^_b_	25.5 ± 2.1^a^_cd_
***Salmonella* Typhimurium**	94.9 ± 0.4^b^_de_	48.1 ± 4.7^a^_a_	36.2 ± 5.5^a^_b_	35.9 ± 11.2^a^_d_
***Salmonella* 1,4,(5),12:i-**	29.1 ± 2.8^b^_a_	99.6 ± 0.6^d^_de_	78.6 ± 5.2^c^_d_	13.5 ± 3.4^a^_abc_
***Salmonella* Virchow**	77.5 ± 1.3^d^_bc_	60.6 ± 4.8^c^_b_	34.0 ± 3.7^b^_b_	21.0 ± 2.0^a^_abcd_

For interpretation, see Table 1.

**Table 7:** Maximum thickness (µm) of twenty-four-hour-old biofilms of several *Salmonella* strains of food origin after exposure to different concentrations of benzalkonium chloride (BZK).

SEROTYPE	Strains Exposed to:			
	Nonexposed	BZK-1000 ppm	BZK-5000 ppm	BZK-10,000 ppm
***Salmonella* Agona**	33.3 ± 14.6^a^_abc_	120.3 ± 44.6^b^_b_	23.7 ± 6.1^a^_abcd_	23.3 ± 9.1^a^_a_
***Salmonella* Anatum**	34.7 ± 4.5^c^_abc_	19.3 ± 2.1^b^_a_	7.7 ± 0.6^a^_a_	22.3 ± 2.5^b^_a_
***Salmonella* Enteritidis**	21.0 ± 4.6^a^_ab_	107.0 ± 40.0^b^_b_	20.3 ± 2.1^a^_abc_	20.3 ± 12.3^a^_a_
***Salmonella* Hadar**	42.3 ± 2.5^b^_bc_	31.0 ± 5.3^ab^_a_	42.7 ± 11.2^b^_d_	22.7 ± 0.6^a^_a_
***Salmonella* Infantis**	25.3 ± 7.5^ab^_ab_	41.3 ± 9.8^c^_a_	23.7 ± 5.7^ab^_abcd_	22.3 ± 4.2^a^_a_
***Salmonella* Kentucky**	35.7 ± 9.2^b^_abc_	31.7 ± 4.0^ab^_a_	17.7 ± 2.5^a^_ab_	21.0 ± 2.7^a^_a_
***Salmonella* Thompson**	22.0 ± 2.7^a^_ab_	31.3 ± 9.3^a^_a_	23.0 ± 4.6^a^_abc_	20.3 ± 1.2^a^_a_
***Salmonella* Typhimurium**	53.3 ± 13.8^b^_c_	16.7 ± 2.9^a^_a_	28.3 ± 2.9^a^_bcd_	22.7 ± 3.2^a^_a_
***Salmonella* 1,4,(5),12:i-**	15.7 ± 2.1^a^_a_	102.0 ± 14.8^b^_b_	38.3 ± 14.2^a^_cd_	24.3 ± 0.6^a^_a_
***Salmonella* Virchow**	30.0 ± 10.2^a^_abc_	28.7 ± 10.0^a^_a_	34.3 ± 2.1^a^_bcd_	21.0 ± 1.7^a^_a_

For interpretation, see Table 1.

**Table 8:** Roughness of twenty-four-hour-old biofilms of several *Salmonella* strains of food origin after exposure to different concentrations of benzalkonium chloride (BZK).

SEROTYPE	Strains Exposed to:			
	Nonexposed	BZK-1000 ppm	BZK-5000 ppm	BZK-10,000 ppm
***Salmonella* Agona**	0.451 ± 0.019^ab^_ab_	0.246 ± 0.028^a^_a_	0.668 ± 0.065^bc^_cd_	0.702 ± 0.170^c^_a_
***Salmonella* Anatum**	0.586 ± 0.068^b^_abcd_	0.439 ± 0.006^a^_bcd_	0.395 ± 0.031^a^_a_	0.703 ± 0.033^c^_a_
***Salmonella* Enteritidis**	0.682 ± 0.048^b^_d_	0.335 ± 0.058^a^_abc_	0.684 ± 0.026^b^_e_	0.648 ± 0.192^b^_a_
***Salmonella* Hadar**	0.644 ± 0.067^b^_d_	0.397 ± 0.008^a^_abcd_	0.676 ± 0.051^b^_e_	0.668 ± 0.028^b^_a_
***Salmonella* Infantis**	0.628 ± 0.031^a^_cd_	0.649 ± 0.142^a^_e_	0.617 ± 0.006^a^_cd_	0.696 ± 0.202^a^_a_
***Salmonella* Kentucky**	0.442 ± 0.060^a^_a_	0.503 ± 0.044^a^_cde_	0.419 ± 0.051^a^_a_	0.699 ± 0.067^b^_a_
***Salmonella* Thompson**	0.567 ± 0.089^b^_abcd_	0.391 ± 0.005^a^_abcd_	0.563 ± 0.047^b^_bc_	0.563 ± 0.003^b^_a_
***Salmonella* Typhimurium**	0.474 ± 0.071^a^_abc_	0.407 ± 0.020^a^_abcd_	0.626 ± 0.020^b^_cd_	0.701 ± 0.016^b^_a_
***Salmonella* 1,4,(5),12:i-**	0.609 ± 0.035^c^_bcd_	0.314 ± 0.075^a^_ab_	0.464 ± 0.027^b^_ab_	0.696 ± 0.019^c^_a_
***Salmonella* Virchow**	0.452 ± 0.031^a^_ab_	0.553 ± 0.060^ab^_de_	0.639 ± 0.022^bc^_cd_	0.726 ± 0.033^c^_a_

For interpretation, see Table 1.

## References

[1] EFSA (European Food Safety Authority) and ECDC (European Centre for Disease Prevention and Control) (2018). The European Union summary report on trends and sources of zoonoses, zoonotic agents and food-borne outbreaks in 2017. EFSA J..

[2] European Commission (2018). The Rapid Alert System for Food and Feed (RASFF), 2017 Annual Report.

[3] EFSA (European Food Safety Authority) and ECDC (European Centre for Disease Prevention and Control) (2017). The European Union summary report on trends and sources of zoonoses, zoonotic agents and food-borne outbreaks in 2016. EFSA J..

[4] González-Machado C., Capita R., Riesco-Peláez F., Alonso-Calleja C. (2018). Visualization and quantification of the cellular and extracellular components of *Salmonella* Agona biofilms at different stages of development. PLoS ONE.

[5] Piercey M.J., Ells T.C., Macintosh A.J., Hansen L.T. (2017). Variations in biofilm formation, desiccation resistance and benzalkonium chloride susceptibility among *Listeria monocytogenes* strains isolated in Canada. Int. J. Food Microbiol..

[6] Buzón-Durán L., Alonso-Calleja C., Riesco-Peláez F., Capita R. (2017). Effect of sub-inhibitory concentrations of biocides on the architecture and viability of MRSA biofilms. Food Microbiol..

[7] Waghmare R.B., Annapure U.S. (2015). Integrated effect of sodium hypochlorite and modified atmosphere packaging on quality and shelf life of fresh-cut cilantro. Food Pack. Shelf Life.

[8] ECHA. European Chemicals Agency Sodium Hypochlorite. https://echa.europa.eu/es/substance-information/-/substanceinfo/100.028.790.

[9] Henriques A.R., Fraqueza M.J. (2017). Biofilm-forming ability and biocide susceptibility of *Listeria monocytogenes* strains isolated from ready-to-eat meat-based food products food chain. LWT Food Sci. Technol..

[10] ECHA. European Chemicals Agency Quaternary Ammonium Compounds, Benzyl-C12–18-alkyldimethyl, Chlorides. https://echa.europa.eu/es/substance-information/-/substanceinfo/100.063.544.

[11] Díez-García M., Capita R., Alonso-Calleja C. (2012). Influence of serotype on the growth kinetics and the ability to form biofilms of *Salmonella* isolates from poultry. Food Microbiol..

[12] Silva P., Goulart L.R., Reis T.F.M., Mendoça E.P., Melo R.T., Penha V.A.S., Peres P.A.B.M., Hoepers P.G., Beletti M.E., Fonseca B.B. (2019). Biofilm formation in different *Salmonella* serotypes isolated from poultry. Curr. Microbiol..

[13] Condell O., Iversen C., Cooney S., Power K.A., Walsh C., Burgess C., Fanning S. (2012). Efficacy of biocides used in the modern food industry to control *Salmonella enterica*, and links between biocide tolerance and resistance to clinically relevant antimicrobial compounds. Appl. Environ. Microbiol..

[14] Corcoran M., Morris D., De Lappe N., O’Connor J., Lalor P., Dockery P., Cormican M. (2014). Commonly used disinfectants fail to eradicate *Salmonella enterica* biofilms from food contact surface materials. Appl. Environ. Microbiol..

[15] Steenackers H., Hermans K., Vanderleyden J., De Keersmaecker S.C.J. (2011). *Salmonella* biofilms: An overview on occurrence, structure, regulation and eradication. Food Res. Int..

[16] Stepanović S., Cirković I., Ranin L., Svabić-Vlahović M. (2004). Biofilm formation by *Salmonella* spp. and *Listeria monocytogenes* on plastic surface. Lett. Appl. Microbiol..

[17] Patel J., Sharma M. (2010). Differences in attachment of *Salmonella enterica* serovars to cabbage and lettuce leaves. Int. J. Food Microbiol..

[18] Vestby L.K., Moretro T., Langsrud S., Heir E., Nesse L.L. (2009). Biofilm forming abilities of *Salmonella* are correlated with persistence in fish meal- and feed factories. BMC Vet. Res..

[19] O’Toole G., Kaplan H.B., Kolter R. (2000). Biofilm formation as microbial development. Annu. Rev. Microbiol..

[20] Norouzi F., Mansouri S., Moradi M., Razavi M. (2010). Comparison of cell surface hydrophobicity and biofilm formation among ESBL-and non–ESBL-producing *Pseudomonas aeruginosa* clinical isolates. Afr. J. Microbiol. Res..

[21] Nesse L.L., Nordby K., Heir E., Bergsjoe B., Vardund T., Nygaard H., Holstad G. (2003). Molecular analyses of *Salmonella enterica* isolates from fish feed factories and fish feed ingredients. Appl. Environ. Microbiol..

[22] Shi X., Zhu X. (2009). Biofilm formation and food safety in food industries. Trends Food Sci. Technol..

[23] Sanchez S., Hofacre C.L., Lee M.D., Maurer J.J., Doyle M.P. (2002). Animal sources of salmonellosis in humans. J. Am. Vet. Med. Assoc..

[24] Weill F.X., Bertrand S., Guesnier F., Baucheron S., Grimont P.A.D., Cloeckaert A. (2006). Ciprofloxacin-resistant *Salmonella* Kentucky in travelers. Emerg. Infect. Dis..

[25] Rodríguez-Melcón C., Riesco-Peláez F., Carballo J., García-Fernández C., Capita R., Alonso-Calleja C. (2018). Structure and viability of 24- and 72-h-old biofilms formed by four pathogenic bacteria on polystyrene and glass contact surfaces. Food Microbiol..

[26] Marin C., Hernandiz A., Lainez M. (2009). Biofilm development capacity of *Salmonella* strains isolated in poultry risk factors and their resistance against disinfectants. Poultry Sci..

[27] Capita R., Buzón-Durán L., Riesco-Peláez F., Alonso-Calleja C. (2017). Effect of sub-lethal concentrations of biocides on the structural parameters and viability of the biofilms formed by *Salmonella* Typhimurium. Foodborne Pathog. Dis..

[28] Sarwari A.F., Magder L.S., Levine P., McNamara A.M., Knower S., Armstrong G.L., Etzel R., Hollingsworth J., Morris J.G. (2001). Serovar distribution of *Salmonella* isolates found in humans. J. Infect. Dis..

[29] Capita R., Alonso-Calleja C., Prieto M. (2007). Prevalence of *Salmonella enterica* serovars and genovars from chicken carcasses in slaughterhouses in Spain. J. Appl. Microbiol..

[30] Wang H., Ding S., Dong Y., Ye K., Xu X., Zhou G. (2013). Biofilm Formation of *Salmonella* serotypes in simulated meat processing environments and its relationship to cell characteristics. J. Food Prot..

[31] Schonewille E., Nesse L.L., Hauck R., Windhorst D., Hafez H.M., Vestby L.K. (2012). Biofilm building capacity of *Salmonella enterica* strains from the poultry farm environment. FEMS Immunol. Med. Microbiol..

[32] Norwood D.E., Gilmour A. (2000). The growth and resistance to sodium hypochlorite of *Listeria monocytogenes* in a steady-state multispecies biofilm. J. Appl. Microbiol..

[33] Holah J.T. (1995). Disinfection of food production areas. Rev. Sci. Tech..

[34] Capita R., Riesco-Peláez F., Alonso-Hernando A., Alonso-Calleja C. (2014). Exposure of *Escherichia coli* ATCC 12806 to sublethal concentrations of food-grade biocides influences its ability to form biofilm, resistance to antimicrobials, and ultrastructure. Appl. Environ. Microbiol..

[35] Rodríguez-Melcón C., Capita R., Rodríguez-Jerez J.J., Martínez-Suárez J.V., Alonso-Calleja C. (2019). Effect of low doses of disinfectants on the biofilm-forming ability of *Listeria monocytogenes*. Foodborne Pathog. Dis..

[36] Poimenidou S.V., Chrysadakou M., Tzakoniati A., Bikouli V.C., Nychas G.J., Skandamis P.N. (2016). Variability of *Listeria monocytogenes* strains in biofilm formation on stainless steel and polystyrene materials and resistance to peracetic acid and quaternary ammonium compounds. Int. J. Food Microbiol..

[37] Tamburro M., Ripabelli G., Vitullo M., Dallman T.J., Pontello M., Amar C.F.L., Sammarco M.L. (2015). Gene expression in *Listeria monocytogenes* exposed to sublethal concentration of benzalkonium chloride. Comp. Immunol. Microbiol. Infect. Dis..

[38] Gilbert P., McBain A.J. (2003). Potential impact of increased use of biocides in consumer products on prevalence of antibiotic resistance. Clin. Microbiol. Rev..

[39] Russell A.D. (2003). Biocide use and antibiotic resistance: The relevance of laboratory findings to clinical and environmental situations. Lancet Infect. Dis..

[40] Moretro T., Vestby L.K., Nesse L.L., Storheim S.E., Kotlarz K., Langsrud S. (2009). Evaluation of efficacy of disinfectants against *Salmonella* from the feed industry. J. Appl. Microbiol..

[41] Murga R., Stewart P.S., Daly D. (1995). Quantitative analysis of biofilm thickness variability. Biotechnol. Bioeng..

